# Roles of RNA Structures in the Genome Translation of (+) Sense RNA Viruses

**DOI:** 10.3390/v17111404

**Published:** 2025-10-22

**Authors:** Guangming Lu, Bethel G. Beyene, Joshua Miguele Camacho, Deepak Koirala

**Affiliations:** Department of Chemistry and Biochemistry, University of Maryland, Baltimore County, Baltimore, MD 21250, USA; glu2@umbc.edu (G.L.); bbeyene1@umbc.edu (B.G.B.); jcamach2@umbc.edu (J.M.C.)

**Keywords:** (+) sense RNA viruses, viral genome translation, internal ribosome entry site (IRES), cap-independent translation enhancer (CITE), ribosomal frameshifting element (FSE), viral RNA structures, viral RNA-protein interactions, virus–host interactions

## Abstract

Positive (+) sense RNA viruses include many important pathogens that exploit noncanonical translation mechanisms to express their genomes within the host cells. Unlike DNA or negative (−) sense RNA viruses, (+) sense RNA viruses can directly function as mRNAs, even though they lack typical features of host mRNAs, such as the 5′ cap structure required for canonical translation initiation. Instead, they exploit structured RNA elements to recruit host translational machinery without the 5′ cap, bypassing the canonical translation initiation mechanism. Prominent examples include internal ribosome entry sites (IRESs) and 3′ cap-independent translation enhancers (3′ CITEs). These RNA modules facilitate translation initiation by recruiting the ribosomal subunits, either directly or through initiation factors, and mediating long-range RNA-RNA interactions. Other regulatory motifs, such as frameshifting signals, allow the ribosome to shift reading frames to regulate protein output. All these RNA elements function through RNA-protein interactions and often utilize host and virus-encoded proteins to hijack the host’s translational apparatus. Over the past several years, various structural biology approaches, including biochemical and enzymatic probing, X-ray crystallography, nuclear magnetic resonance (NMR) spectroscopy, and cryogenic electron microscopy (cryo-EM), have revealed the unique structural roles of these viral RNA elements and their protein complexes. Although a few structures of IRES and CITE domains have been solved through these methods, the structures of these RNA elements and their structure-function relationship have remained largely unknown. This review discusses the current understanding of translation-related RNA structures in (+) sense RNA viruses, the critical RNA-protein interactions they mediate, and various structural biology approaches used to study them. Since the genome of these viruses serves as a template for two mutually exclusive virological processes, namely genome translation and replication, the review also discusses how viruses can utilize RNA structure-based strategies to regulate the switch between genome translation and replication, highlighting future directions for exploring these fundamental virological processes to develop antiviral therapeutics able to combat diseases caused by these pathogens.

## 1. Introduction

Viruses are classified into several groups based on their genome type and replication strategy. The Baltimore classification divides viruses into five broad groups: double-stranded DNA (dsDNA), single-stranded DNA (ssDNA), double-stranded RNA (dsRNA), positive (+) sense single-stranded (ss) RNA, negative (−) sense ssRNA, and reverse-transcribing viruses [1,2]. Among these groups, (+) sense RNA viruses include many medically important families, including zoonotic viruses such as those belonging to the *Picornaviridae*, *Flaviviridae*, and *Coronaviridae* families and plant viruses such as those belonging to the *Potyviridae* and *Tombusviridae* families [3,4,5,6,7]. In contrast to DNA viruses or (−) sense RNA viruses, (+) sense RNA viral genomes can directly serve as an mRNA upon entry to the host cell, allowing immediate translation of viral proteins by the host translation machinery [8,9,10].

**Figure 1:**
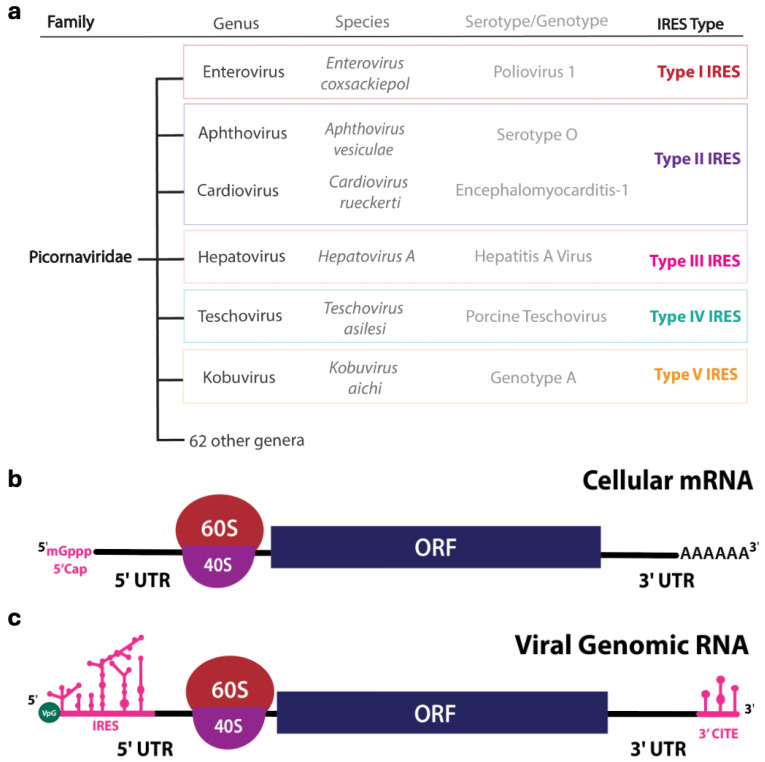
Types of Picornaviral IRESs and organization of cellular mRNA (cap-dependent translation) and a (+) sense RNA viral genome. (**a**) Types of IRESs found in members of the *Picornaviridae* family demonstrate IRES diversity among various (+) sense RNA viruses. However, this IRES classification extends beyond picornaviruses. For instance, type IV IRESs are also present in Hepatitis C Virus, which belongs to the Flaviviridae family. (**b**) Organization of a typical eukaryotic mRNA, which contains a 5′ m^7^G cap and 3′ poly(A) tail, both of which protect and activate mature RNA (mRNA) for translation. (**c**) Organization of a typical (+) sense RNA viral genome, which promotes a cap-independent translation mechanism in host cells through unique RNA elements within its viral genome. Along with the attachment of VPg, viral genomic RNA contains *cis*-acting elements such as an IRES in the 5′ UTR and 3′ CITEs in the 3′ UTR, enabling interaction with host initiation factors and the ribosome for viral protein translation.

Among several families of (+) sense RNA viruses, the *Picornaviridae* family with 68 genera and 159 species includes the most significant number of human and animal pathogens (Figure 1a), such as Enteroviruses, Hepatoviruses, Cardioviruses and Aphthoviruses [10,11,12]. Unlike host mRNAs (Figure 1b), the genomes of (+) sense RNA viruses typically do not contain a 5′ cap (7-methylguanosine, m_7_G) but often carry a viral protein VPg (viral protein genome-linked) covalently attached to the 5′ end (Figure 1c) [13,14,15]. These viruses must therefore evolve mechanisms for ribosome recruitment that bypass the canonical mRNA translation initiation, which relies on the 5′ cap [16,17]. Thus, they frequently harbor structured RNA elements that mimic or replace the host mRNA translation process, especially the most critical initiation steps. Notably, (+) sense RNA picornaviruses tightly pack multiple functions into their limited-sized genome (typically less than 10 kb), using RNA structures that regulate multiple virological processes such as translation, new RNA synthesis, and genome packaging [10,18]. For example, *cis*-acting replication elements (*cre*) in the RNA genome of enteroviruses can mediate and regulate both replication and translation by bringing the genome’s 5′ and 3′ ends together and recruiting several viral and host protein factors for each mechanism [19,20,21].

**Figure 2:**
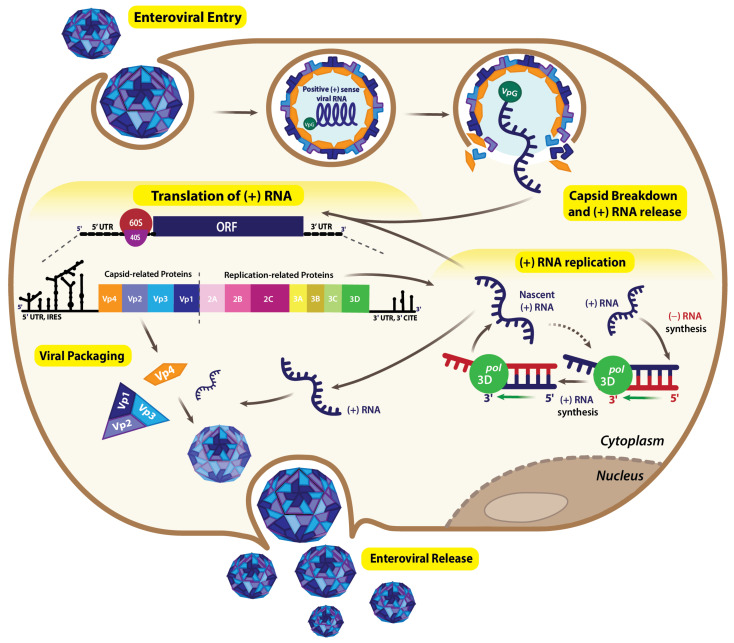
Life cycle of a typical (+) sense RNA virus. The virus is internalized into the host cell via endocytosis, releasing its (+) sense RNA genome into the cytoplasm. The genome directly functions as mRNA, enabling the immediate translation of essential viral proteins by exploiting the host’s ribosomes, amino acids, and other necessary cellular machinery. Translation produces a single polyprotein that, upon proteolysis, produces capsid-related proteins (Vp1, Vp2, Vp3, and Vp4), which assist in viral packaging and encapsidation, enabling further enteroviral infection. Replication-related proteins (2A, 2B, 2C, 3A, 3B, 3C, and 3D) are also generated during proteolytic processing. With the 3D protein acting as a polymerase, a (−) sense RNA is formed using (+) sense genomic RNA as a template. Subsequently, this intermediate (−) RNA serves as a template to synthesize the (+) sense genomic RNA again, which can then undergo translation and replication or be packaged into new virions.

The lifecycle of a typical (+) sense RNA virus (Figure 2) begins with the virus particle attaching to the host cell surface and entering the cell through membrane fusion or endocytosis [22]. Once in the host cytoplasm, the virus releases its positive-sense genome, which the host’s ribosomes directly translate to produce genome-encoded proteins. Viral translation usually produces a polyprotein (in picornaviruses and flaviviruses) or sets of structural and nonstructural proteins (in coronaviruses) [23]. The nonstructural proteins include an RNA-dependent RNA polymerase (RdRp) that catalyzes the genomic RNA replication [24]. This often involves synthesizing a complementary (−) sense strand, which then serves as a template for synthesizing the new (+) sense genomic RNA. Because the same RNA sense is a template for two mutually exclusive processes, replication and translation, these two processes must be coordinated, and (+) sense RNA viruses must temporally regulate the use of their genomes for translation and replication. Typically, the incoming genome is first translated to produce nonstructural proteins, including replication factors, before switching to serve as a template for RNA synthesis [25,26]. Finally, the newly synthesized genomic RNAs are packaged with capsid proteins to form new virions, which exit the cell by lysis or vesicle formation [22]. While both translation and replication of viral genomes depend on various host factors and involve multiple steps, this review focuses on the fundamentals of (+) sense RNA genome translation mechanisms in eukaryotic host cells, with particular attention to recent advancements in understanding the structural biology of these unique RNA elements.

**Figure 3:**
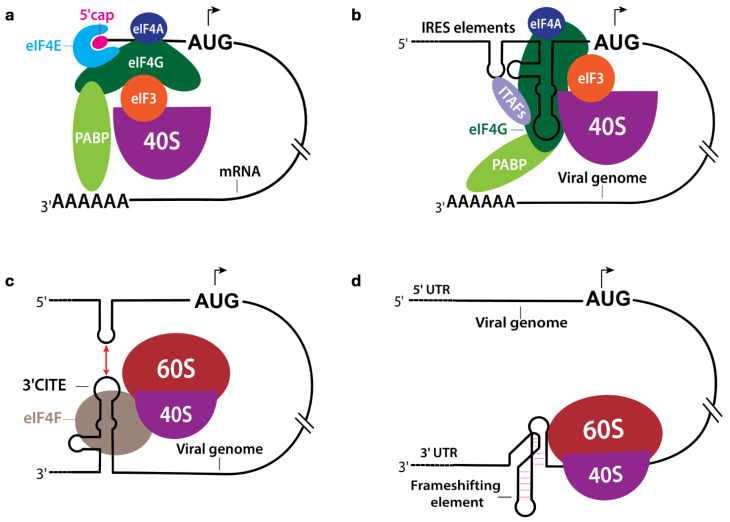
Mechanisms of translation initiation and regulation in virus-infected eukaryotic cells. (**a**) Cap-dependent translation in uninfected eukaryotic cells depends on the binding of initiation factors and a poly-A binding protein. The eIF4E in the eIF4F complex (comprising eIF4E, eIF4A, eIF4G, and eIF3) attaches to the 5′ m7G cap to facilitate the attachment of the eIF-bound 40S ribosomal subunit to the mRNA for translation. Cap-independent translation in (+) sense RNA involves using (**b**) IRES or (**c**) 3′ CITE to recruit host factors. Utilizing the IRES or 3′ CITE bypasses the need for a cap, as these elements can bind to eIFs and interact with the host ribosome without this feature. (**d**) Frameshifting elements embedded in the (+) sense RNA can influence ribosomal activity, which regulates the levels of specific viral proteins being translated that may be required at a particular stage of the viral lifecycle.

In uninfected eukaryotic cells, most mRNAs have an m^7^G cap at their 5′ ends, which is recognized by the eukaryotic initiation factor 4E (eIF4E), a subunit of the eIF4F complex (composed of eIF4E, eIF4G, and eIF4A) [17,27]. Upon binding with the cap via eIF4E, the eIF4F complex recruits the 43S pre-initiation complex (40S ribosomal subunit bound to eIF1, eIF1A, eIF3, eIF5, and the ternary complex eIF2–Met-tRNA-iGTP) [17]. The 43S complex then scans along the 5′ untranslated region (UTR) from the cap to find an authentic AUG start codon in a favorable context [28,29]. Upon recognizing the start codon by the 43S complex, GTP hydrolysis and the release of initiation factors promote the joining of the 60S subunit to form an 80S ribosome initiation complex ready for elongation [30]. This cap-dependent scanning mechanism (Figure 3a) is highly efficient for typical cellular mRNAs, but can be inhibited or suppressed during stress conditions or viral infections by disrupting the normal function of one or more components of the translation machinery [31].

Many (+) sense RNA viruses bypass the cap-dependent initiation mechanism by using *cis*-acting RNA elements in their genomes, such as internal ribosome entry sites (IRESs) and 3′ cap-independent translation enhancers (3′ CITEs) (Figure 3b,c). These elements usually contain structured RNA domains that can recruit ribosomes directly or via translation initiation factors. For example, IRES elements within the 5′ UTR of enteroviral genomes recruit the 40S subunit through complex interactions with multiple initiation factors and IRES trans-acting factors (ITAFs), forming initiation complexes without requiring a 5′ cap [16,32,33,34,35,36,37]. Similarly, 3′ CITEs within the 3′ UTR of several plant viruses bind initiation factors (often the eIF4F complex) or ribosomal subunits and interact with the 5′ end through long-range base-pairing, facilitating translation initiation [38,39,40]. Such cap-independent mechanisms allow viral protein synthesis even when host cap-dependent translation is downregulated during infection or stress. Besides IRESs and 3′ CITEs that directly regulate the translation initiation of viral genomes, some RNA structures, such as programmed ribosomal frame-shifting elements, are involved during the translation elongation to control the abundance of specific viral proteins at different stages of the viral life cycle [41,42,43] (Figure 3d). Furthermore, some viruses use small proteins, such as VPg, to covalently attach to their genomic RNAs to mimic the 5′ cap structure, which can interact with eIF4E or other host factors to initiate translation [44]. Therefore, by exploiting these specialized RNA structures in their genomes, (+) sense RNA viruses can effectively hijack the host translational apparatus to ensure efficient viral protein production in host cells.

## 2. RNA Structures Associated with Viral Genome Translation

### 2.1. Internal Ribosome Entry Site (IRES)

IRESs are *cis*-acting RNA elements found in the genomes of many (+) sense RNA viruses and some cellular RNAs that promote cap-independent translation initiation by directly recruiting the ribosome to internal sites within viral genomes or cellular mRNAs [45]. Such a mechanism bypasses the need for a 5′ cap structure essential in the canonical translation mechanism of cellular mRNAs. Therefore, IRESs are crucial for efficient translation when cap-dependent initiation is compromised, like during viral infection or cellular stress [36,46,47,48]. IRES-mediated translation is often regulated by IRES ITAFs, which can either enhance or suppress IRES activity depending on the cellular environment [49,50]. While IRESs are common among (+) sense RNA viruses, including members of the *Picornaviridae*, *Flaviviridae*, and *Dicistroviridae* families, they show considerable sequence and structural diversity despite their similar role in viral genome translation. Including those found in the *Picornaviridae* family, which is the largest group of (+) sense RNA viruses and includes many viruses that infect humans, IRESs can be categorized into five main types based on their predicted RNA secondary structures, requirements for initiation factors, and differences in how ribosomes are recruited for translation initiation (Table 1).

**Figure 4:**
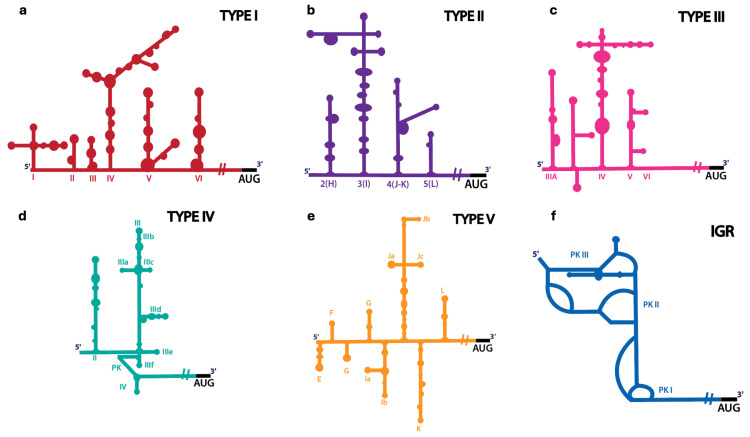
Various types of IRESs and their predicted secondary structures. The subdomains and major secondary structural features are labeled. Types I–III IRESs (**a–c**) are prevalent among members of the *Picornaviridae* family. The type IV IRESs (**d**) were initially found in members of *Flaviviridae*, such as HCV and CSFV, but they were later identified in the porcine teschovirus, a member of the *Picornaviridae*. Type V IRES (**e**) was recognized in Aichi viruses. The Intergenic region (IGR) IRES (**f**) is found in viruses such as cricket paralysis virus (CrPV), with some structural and ribosome recruitment features similar to type IV IRESs.

Type I: These are found in enteroviral genomes and are among the first IRESs identified in *Enterovirus coxsackiepol* (formerly termed as poliovirus, PV) and enterovirus 71 (EV71), belonging to the *Enterovirus alphacoxsackie* species [16,51]. The IRES segment typically spans approximately 450 nucleotides and has five structural domains (II to VI, Figure 4a). Translation initiation by type I IRESs requires multiple initiation factors, including eIF1A, eIF2, eIF3, eIF4A, eIF4B, eIF4G and eIF5B [52,53,54] as well as ITAFs such as PCBP2 [53,54,55], PTB [54,56], La [57], and Unr [58]. Each IRES domain tends to bind to these specific factors that promote the recruitment of the 40S ribosome. Initiation involves ribosomal scanning by the 43S preinitiation complex to find the authentic AUG start codon [53]. Unlike the canonical mechanism, they do not require eIF4E and utilize the cleaved form of eIF4G (residues 737-1116) [51,59]. The domain V of type I IRES has been shown to interact with the central domain of eIF4G, which recruits eIF4A and causes downstream conformational changes that may enable the 43S preinitiation complex to bind [59].

Type II: These are typically found in members of the *Cardiovirus* and *Aphthovirus* genera, such as Cardiovirus rueckerti (formerly named encephalomyocarditis virus, EMCV) and *Aphthovirus* vesiculae (formerly named foot-and-mouth disease virus, FMDV) [60,61]. Type II IRES is also found in calicivirus genomes [62]. Similar to Type I IRESs, Type II IRESs are approximately 450 nucleotides long [63] and contain modular elements whose domains are designated H to L (Figure 4b) [64]. These elements directly recruit eIF4G and eIF4A via a conserved J-K domain, promoting ribosome loading without eIF4E or scanning factors such as eIF1 and eIF1A [65]. However, a study has shown that EMCV IRES can directly bind to the 40S subunit without the assistance of eIF4G/4A, but its start codon positioning and assembly efficiency of the 48S pre-initiation complex are higher with the help of factors [66]. Moreover, studies on FMDV and Avian Calicivirus IRESs demonstrate that initiation efficiency and site preference are dynamically regulated by canonical initiation factors and ITAFs [54,67,68,69]. In particular, PTB, Gemin5, Sam68, or ITAF45 can either stimulate or repress initiation at specific sites, often through binding to distinct structural domains within the IRES and inducing conformational remodeling that alters factor recruitment [70,71,72]. Thus, the interplay between initiation factors and ITAFs, together with structural plasticity of the type II IRES, can fine-tune ribosomal recruitment and start codon selection during cap-independent translation.

Type III: Only the Hepatovirus A (formerly named hepatitis A virus, HAV) has been considered in this category, so some studies and previous reviews have highlighted only four types of viral IRESs [29,73,74]. The HAV IRES spans approximately 735 nts in the 5′ UTR and comprises six structured domains (designated I to VI) [75,76]. Although HAV IRES requires all six domains for optimal function, domains III–VI (Figure 4c) have been shown to effectively drive internal initiation, nearly reaching the level of the IRES containing all six domains [76,77]. The HAV IRES requires the complete eIF4F complex, including eIF4E [78], distinguishing it from other picornaviral IRES types. Moreover, unlike type II IRESs, which function with a cleaved form of eIF4G, HAV IRES requires a full-length eIF4G [73,78]. Mechanistically, the proposed initiation proceeds as follows: the IRES structure is recognized by eIF4E binding, which recruits eIF4G; eIF4A-mediated helicase activity helps to resolve local secondary structure near the initiation codon; this allows for recruitment of the 43S pre-initiation complex [79]. Subsequently, the 60S subunit joins to form the 80S ribosome and begins elongation. HAV IRES activity is further modulated by liver cell-specific enhancers or auxiliary factors (including recently found PDAP1), which increase translation efficiency in hepatocyte extracts relative to non-hepatic cells [80].

**Table 1:** Classification of six types of IRESs based on their predicted secondary structures and their requirements for eukaryotic initiation factors (eIFs) for translation.

IRES Types	Length	Virus	Secondary Structure	eIFs	ITAFs
Type I	~450 nts	*Enterovirus coxsackiepol*, *Enterovirus alpharhino*, *Enterovirus betarhino*, *Enterovirus cerhino*, *Caliciviruses*	Five modular domains (II to VI) [81]	eIF1A, eIF2, eIF3, eIF4A, eIF4G, eIF4B, eIF5B [52,53,54]	PCBP2 [53,54,55], PTB [54,56], La [57], Unr [58], Sam68 [82]
Type II	~450 nts	*Cardiovirus rueckerti*, *Aphthovirus vesiculae*, *Caliciviridae*	Five modular domains (H to L) [64,83,84]	eIF1, eIF1A, eIF2, eIF3, eIF4G, eIF4A [67,68,85,86]	PTB [70], PTB + ITAF45/EBP1 [68], Gemin5 [87], Sam68 [88]
Type III	~700 nts	*Hepatovirus A*	Five modular domains (II to VI) [76,77]	eIF2, eIF3, eIF4A, eIF4G, eIF4B, eIF4E [73,78,79]	PDAP1 [80], PTB [89], PCBP2 [90,91], La [92]
Type IV	~300 nts	Hepatitis C virus, Classical swine fever virus, Penguin megirivirus, Ruddy turnstone calicivirus	Compact structure with pseudoknots (II to III) [93,94]	eIF1A, eIF2, eIF3, eIF5B [95,96,97,98,99,100,101]	La [57,102], hnRNP L [103], hnRNP D [104]
Type V	~450 nts	*Kobuvirus aichi*, Calicivirus	Eight modular domains (E to L) [105]	eIF2, eIF3, eIF4A, eIF4G [105]	DHX29 [105], PTB [105]
IGR	~200 nts	Cricket paralysis Virus	Three nested pseudoknots (PKI to PKIII) [106]	NA	NA

Type IV: Although these IRESs have been historically discussed in the context of viruses like the hepatitis C virus (HCV) and the classical swine fever virus (CSFV), which are members of the *Flaviviridae* [107,108,109], members of the *Picornaviridae* family have also been shown to contain type IV IRESs. Some recently discovered examples include the penguin megirivirus [110] and Seneca virus Cetus [111], demonstrating that these IRES types are widespread in many viruses affecting even marine animals such as penguins and porpoises. The type IV IRES has an intricate and well-folded RNA structure comprising multiple stem loops and pseudoknots (Figure 4d) [94,112,113]. The HCV IRES can directly bind the 40S ribosomal subunit and position the start codon in the P site without requiring eIF4F, eIF4A, or eIF4B, a process reminiscent of prokaryotic translation initiation [95]. Moreover, studies have shown that IRES domain II facilitates 80S ribosome formation by promoting eIF5-induced GTP hydrolysis and eIF2–GDP release, thereby driving subunit joining [96]. Remarkably, under stress conditions when eIF2 is phosphorylated and inactivated, the HCV IRES can bypass the canonical eIF2-dependent initiation and instead use an alternative pathway involving only eIF3 and eIF5B [98]. However, its function is regulated by multiple specific ITAFs, such as La autoantigen, hnRNP L and hnRNP D [57,102,103,104]. Upon binding with the 40S ribosomal subunit and eIF3, type IV IRES aligns the AUG start codon into the P site through base pairing with 18S rRNA [114]. Subsequently, the recruitment of eIF2-GTP-Met-tRNAᵢ and the joining of the 60S subunit, mediated by eIF5B, complete the translation initiation process [115,116,117].

Type V: The IRESs in this group are believed to be of chimeric evolutionary origin, exhibiting the structural features of both type I and II IRESs. Examples of type V IRESs include those found in *Kobuvirus aichi* (formerly named Aichivirus, AV) [105]. These IRESs possess eight structural domains, designated E to L (Figure 4e) and require eIF2, eIF3, eIF4A, and the middle domain of the eIF4G (residues 736-1115). Notably, the critical dependency on the DEAH-box RNA helicase DHX29 distinguishes Type V IRESs from other picornavirus IRES classes. The dependency on DHX29 likely indicates the need to unwind stable RNA structures, such as hairpins, which can likely sequester the start codon [105].

IGR: Another distinct class of IRESs is intergenic region (IGR) IRESs (Figure 4f), which represent a unique class of viral IRESs found in the *Dicistroviridae* family [118], such as Cricket paralysis virus (CrPV) [119,120]. Unlike most viral IRESs in the 5′ UTR, IGR IRESs reside between two open reading frames. They directly assemble elongation-competent 80S ribosomes without canonical initiation factors or Met-tRNA [119,121,122,123]. Structural and biochemical studies have revealed that the IGR IRES adopts a tRNA-mimicking pseudoknot architecture, comprising three nested pseudoknots (PKI to PKIII), with PKI mimicking an anticodon stem–loop base-paired to mRNA and occupying the ribosomal P site [106,122,123,124]. Mechanistically, the IGR IRES first binds to the 40S subunit without eIFs and subsequently recruits the 60S subunit to form an 80S complex. While the PKI domain occupies the ribosomal P site, parts of PKII and PKIII occupy regions normally occupied by E-site and A-site tRNAs, thus positioning the start codon for translation elongation. Although eIF1, eIF1A, and eIF3 can inhibit subunit joining in vitro, the IGR IRES can bypass these inhibitory effects via direct 80S ribosome binding [119,125]. Notably, in Halastavi arva virus (HalV), which lacks domain 2, the IGR IRES employs an even simpler mechanism—directly binding 80S ribosomes with PKI pre-positioned in the P site—allowing the A site to immediately accept the first sense codon without the need for eEF2-mediated translocation [126]. Recent in-cell studies using infectious CrPV clones confirm the physiological relevance of specific IRES structural elements, showing that mutations disrupting pseudoknot formation can impair viral translation and prevent virus production in Drosophila S2 cells. These studies highlight that IGR IRES function is not only a product of its unique structure but also of dynamic interactions with the ribosome in a cellular context [122]. Recent bioinformatics and functional screens revealed that dicistro-like IRES elements are widespread, with about 32% of over 4700 genomes containing putative IRESs, including multiple IRESs or IRESs embedded within ORFs [127]. These elements bind ribosomes directly and support internal translation in vitro and in vivo. Similar factor-less IRES-like structures were also identified in non-dicistrovirus genomes, including *Tombusviridae* and *Narnaviridae* families, highlighting the diversity of this mechanism in many viruses [127]. Taken together, these findings reveal the mechanistic strategy by which IGR IRESs recruit ribosomes and initiate translation independently of canonical eukaryotic translation initiation factors, providing insights into their molecular architecture and physiological function.

### 2.2. 3′ Cap-Independent Translation Enhancer (3′ CITE)

The 3′ cap-independent translation enhancer (3′ CITEs) are structured RNA elements typically found in the 3′ UTRs of many (+) sense RNA viruses. To date, they have only been identified in plant viruses, especially those in the *Tombusviridae* and *Luteoviridae* families [128,129,130]. These elements play key roles in promoting efficient translation of viral RNAs that lack typical eukaryotic mRNA features, such as the 5′ cap and poly(A) tail [131,132,133]. The discovery of 3′ CITEs demonstrates an alternative and convergently evolved strategy for cap-independent translation, highlighting the adaptability of viral genomes to exploit host translation machinery, regardless of the viral sources, infected hosts or location of these RNA domains within their genomes (5′ or 3′ UTRs).

**Figure 5:**
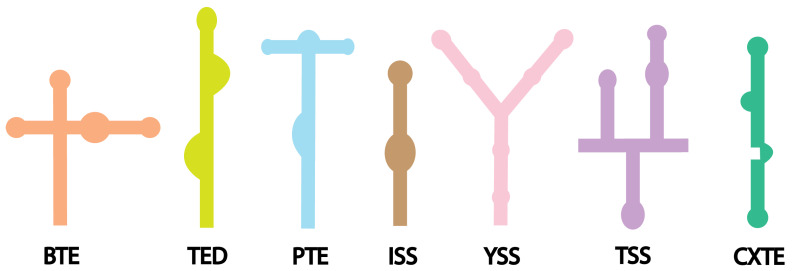
Various types of 3′ CITEs and their predicted secondary structures. BTE was identified in Barley yellow dwarf virus [135]. TED was among the first 3′ CITEs discovered in the satellite Tobacco necrosis virus [128]. PTE and ISS are found in panicum mosaic virus and maize necrotic streak virus and bind eIF4E through a flipped-out guanine within their structures [136,137]. YSS has been reported in Tomato bushy stunt virus and tombusviruses [131,138]. They typically contain three stem-loops essential for function. TSS are characteristic of Turnip crinkle virus and fold into intricate structures containing pseudoknots [139,140]. The CXTEs have complex stem-loop structures and are named based on the geographical isolate of the Cucurbit aphid-borne yellows virus, with CXTE originating from Xiajiang [141].

Based on their predicted RNA secondary structures and how initiation factors or ribosome recruitment occur, at least seven distinct classes of 3′ CITEs have been identified (Figure 5 and Table 2). Each class presents unique secondary structures and the mechanisms of action [40]. These elements boost viral translation mainly by recruiting host translation initiation factors (eIFs) to the 3′ end of the viral RNA and then helping deliver them to the 5′ end. The mechanistic model of CITE-mediated translation often involves long-distance RNA-RNA or RNA-protein interactions to circularize the viral genome (Figure 3c). Such a circularization mimics the closed-loop structure in capped and polyadenylated eukaryotic mRNAs, enhancing ribosome recruitment and initiation efficiency [134].

Different 3′ CITE classes utilize specific molecular interactions to enhance translation. For example, the barley yellow dwarf virus-like translation element (BTE) adopts a relatively simple branched hairpin structure that directly interacts with the eIF4G subunit of the eIF4F complex. Structural and biochemical studies have confirmed that the BTE-eIF4G interaction is vital for its enhancer activity [135,142,143,144,145]. Conversely, other 3′ CITEs, such as the panicum mosaic virus-like translational enhancer (PTE) [146], the translation enhancer domain (TED) [128,147], the Y-shaped structure (YSS) [131,138,148], and the I-shaped structure (ISS) [40,134,137,149], mainly interact with the eIF4E subunit. CXTE (X representing the Xiajiang isolate) represents a more complex system; it was first identified in cucurbit aphid-borne yellows virus (CABYV) and later in Melon necrotic spot virus (MNSV). It is a modular and transferable architecture with an intricate stem loop structure that binds eIF4G [141,150]. Some classes of 3′ CITEs display additional functional features. For instance, T-shaped structure (TSS) elements have been shown to bind directly to ribosomal subunits, suggesting a more immediate role in translation initiation [139,151]. Additionally, many 3′ CITEs participate in long-range interactions with complementary sequences in the 5′ UTR, forming kissing-loop structures that facilitate the recruitment and transfer of initiation factors from the 3′ to the 5′ end. A well-known example is the PTE of panicum mosaic virus (PMV), which forms a kissing-loop interaction with the 5′ UTR to promote genome circularization and efficient ribosome assembly [40,136].

**Table 2:** Classification of seven types of 3′ CITEs based on their predicted secondary structures and their interactions with eukaryotic initiation factors (eIFs) for translation. These RNA elements have so far been identified in the plant viruses belonging to the *Tombusviridae* and *Luteoviridae* families.

3′ CITEs	Length	Virus	Secondary Structure	Roles
BTE	~100 nts	Barley Yellow Dwarf Virus [135]	Multiple stem loops [142]	Interacts with eIF4G [144,152]
TED	~120 to150 nts	Satellite *Tobacco Necrosis Virus* [128]	Complex structure, multiple stem loops [128]	Interacts with eIF4E [38]
PTE	~100 nts	Panicum Mosaic Virus [136], Pea enation mosaic virus [153]	Pseudoknot containing structure [153,154]	Interacts with eIF4E, promotes RNA circularization [153]
ISS	~60 nts	Tombusviruses [134], Maize necrotic streak virus [137]	Complex stem loop structure with multiple bulges [134,137]	Interacts with eIF4E [134,155]
YSS	~130 to 150 nts	Tomato bushy stunt virus [131], Tombusvirus [138]	Y-shaped three-way junction architecture [131]	Interacts with eIF4E [153,156]
TSS	~100 nts	Turnip crinkle Virus [139]	T-shaped structure, tRNA-like [139]	Interacts directly to ribosomal subunits, promotes RNA circularization [140]
CXTE	~55 nts	Cucurbit aphid-borne yellows virus [141], Melon Necrotic Spot Virus [150]	Complex structure with multiple stem loops [150]	Interacts with eIF4G [141]

Notably, 3′ CITEs are often functionally modular and can be transferred between viruses through recombination events [129]. Such mobility emphasizes their evolutionary importance and indicates a role in viral adaptation, such as evading host defenses or expanding host range [157]. Despite several differences in structural and mechanistic strategies to ensure efficient translation, 3′ CITEs act as functional equivalents of the 5′ cap and poly(A) tail in plant viruses. Such sequence diversity and evolutionary flexibility of 3′ CITES also highlight the advanced molecular strategies used by the plant viruses to hijack host translational machinery and maximize viral protein production.

### 2.3. Other Regulatory RNA Elements in Viral Translation

IRESs and 3′ CITEs are typically involved in translation initiation; however, many (+) sense RNA viruses utilize structured RNA elements, such as programmed ribosomal frameshifting or readthrough signals, during translation elongation to produce multiple proteins from a single RNA [29]. A well-known example is the programmed -1 ribosomal frameshifting element (FSE) in coronaviruses and retroviruses, which is mediated by a heptanucleotide “slippery” sequence and a downstream pseudoknot structure that causes the ribosome to slip back by one nucleotide [158,159]. Such ribosomal frameshifts help balance the production and abundance of specific viral proteins by altering the reading frame during the translation elongation step. For instance, maintaining the ratio of RdRp to structural proteins is essential for balancing the viral life cycle′s translation, replication, and packaging steps [160,161,162]. While there is no consensus structure of the FSEs, they are typically compact pseudoknot or hairpin structures with high mechanical resistance to ribosome unwinding, which causes a pause to trigger the frame change [163]. Additionally, some viruses employ internal termination-reinitiation signals or programmed +1 frameshifts to regulate gene expression [161]. While detailed structures of these frameshift stimulators are emerging, the core principle remains that these RNA structures are very dynamic, can adopt different conformations, and act as checkpoints to switch translational reading frames, thereby controlling the viral protein stoichiometry in the host cells. Classic studies have shown that viral frameshift pseudoknots and stem–loop structures can adopt multiple competing conformations, and this structural plasticity is directly linked to −1 programmed ribosomal frameshifting (PRF) efficiency [42,163,164,165].

Several other viral or host-derived RNA elements also play crucial roles in translation regulation. For example, the termination–reinitiation system in caliciviruses depends on a short RNA motif called the termination upstream ribosome binding site (TURBS), which base-pairs with 18S rRNA and allows the post-termination 40S subunit to stay engaged and reinitiate translation at a downstream AUG [166]. Another example is the 3′ tRNA-like structure (TLS) found in plants and some viral RNAs, which mimics authentic tRNAs, can be aminoacylated by host tRNA synthetases, and thereby contributes to translation efficiency, RNA stability, and replication control [29]. Similarly, the Gamma-interferon-Activated Inhibitor of Translation (GAIT)and GAIT-like elements, initially identified in cellular mRNAs, form structured motifs in the 3′ UTR that recruit host inhibitory complexes to repress translation initiation. Viruses such as SARS-CoV-2 contain GAIT-like motifs, indicating they can hijack this host regulatory mechanism to adjust their gene expression [167].

## 3. Protein-RNA Interactions in Translation-Associated Complexes

RNA viruses hijack host cellular machinery through specific interactions between viral RNA structures and the viral or host proteins. IRES elements frequently engage a subset of canonical eukaryotic initiation factors (eIFs) and various ITAFs, predominantly a group of host RNA-binding proteins (Table 3). For instance, the poliovirus IRES activity depends on polypyrimidine tract-binding protein (PTB) [47,56], poly(C)-binding protein 2 (PCBP2) [47,168,169,170], and poly(A)-binding protein (PABP), in addition to eIF4G [59] and eIF4A [171]. For picornaviral IRESs, PTB interacts with polypyrimidine tracts within the IRESs [172] and likely induces RNA structural remodeling to facilitate eIF4G positioning [56]. PCBP2 binds to the central domain of type I and III IRESs, enhancing translation activity perhaps by constraining the dynamic regions of the IRES and facilitating the recruitment of other ITAFs and eIFs [168,169,170]. Although the IRES body directly contacts the 40S and positions the AUG into the P site for pairing with the eIF2·GTP·Met-tRNAiMet complex, HCV-type IRESs bind eIF3 through junction IIIabc. Notably, structural data show that the IRES-eIF3 interaction can displace eIF3 from its ribosomal binding site, thus reconfiguring the initiation complex [99,117,173,174,175]. Conversely, *Dicistroviridae* IRESs are distinctive because they bypass the requirement for eIFs; their RNA pseudoknot directly binds to the ribosomal decoding sites to recruit the ribosome [123,176].

Similarly, 3′ CITE functions depend on their interactions with viral and host proteins, though different CITE types exhibit distinct binding mechanisms with translation initiation factors. For example, the TED element of STNV directly interacts with eIF4F to promote translation initiation, while the YSS of tombusvirus cooperatively engages eIF4E and eIF4G to enhance the ribosome recruitment [38,156]. These interactions mimic cellular mRNAs’ canonical cap-dependent translation strategy, through mechanisms that bypass the 5′ cap [144,152,184]. Additionally, PABP often plays a role in several of these non-canonical translation systems. For example, plant viral systems use CITEs that frequently involve PABP-mediated mechanisms to compensate for the absence of a 5′ cap structure, which otherwise interacts with the 3′ end of mRNAs via poly(A) tail and PABP complex [141,152,185].

Many viral RNAs also interact with virus-encoded proteins, playing vital roles in the viral life cycle. A notable example is the VPg protein in picornaviruses and caliciviruses. VPg is covalently attached to the 5′ end of the viral RNA genome via a phosphodiester bond and modulates translation through interactions with host initiation factors such as eIF4E [14,15,186]. In a separate context, it also acts as a primer for viral RNA replication [187,188]. In caliciviruses, VPg binds to eIF4E, effectively replacing the function of the usual mRNA cap structure [189]. In enteroviruses, VPg is crucial for RNA replication: *cre* forms a stem-loop in the viral genome that guides the uridylation of VPg, initiating (−) sense RNA synthesis, which is the first step in (+) sense viral genome replication [21,190,191]. RNA-protein interactions can also serve as molecular switches that coordinate viral genome translation and replication [66,192]. Many viruses contain specialized RNA sequences, called *cis*-acting replication elements, that can recruit both host cellular proteins and viral RNA polymerase [19]. These elements create a regulatory network: when bound by replication machinery, they can suppress translation through the nearby IRES domains, allowing the virus to shift from protein synthesis to genome replication [193]. This temporal regulation ensures efficient viral production by preventing simultaneous translation and replication on the same RNA template [194]. Table 3 above summarizes key host factors (ITAFs) facilitating this coordinated process.

## 4. Structural Studies of Translation-Related RNAs

The diversities among the IRES and 3′ CITE-mediated mechanisms reflect various evolutionary strategies viruses have developed to simplify cap-independent translation of their (+) sense RNA genomes. These elements allow viral genomes to bypass host translational controls and maintain protein synthesis even during a global shutdown of cap-dependent translation, supporting viral propagation, host adaptation, and pathogenicity. To perform their functions, IRES and 3′ CITE elements exhibit complex folds that enable the precise spatial arrangement of functional RNA motifs, which are essential for interactions with ribosomes and initiation factors. For example, the HAV IRES contains a three-way junction stabilized by an adenine-rich loop [195], similar to the J-K domains in EMCV IRES [65], indicating a conserved mechanism for recruiting eIF4G/eIF4A [195]. Recent cryo-EM analyses of the CrPV IRES have shown that it forms a compact fold with multiple pseudoknot structures that directly interact with the 40S ribosomal head and the P site. Instead of using host initiation factors or an upstream AUG to form a 48S preinitiation complex, the CrPV IRES positions a pseudoknot into the P site and employs built-in mimicry to initiate translation, with eEF2-mediated translocation steps required to transition into an elongation-competent 80S ribosome [196,197]. Similarly, the crystal structures of Saguaro cactus virus (SCV) and Pea enation mosaic virus 2 (PEMV2) 3′ CITE domains illustrated how an uncapped viral RNA domain mimics the 5′ cap to recruit the host cap-binding protein, eIF4E [198,199]. While gaining insights into the three-dimensional structure of viral RNA elements and their complexes is crucial to advancing our understanding of viral translation processes [41,151,164,170,196,200], only a handful of high-resolution structures have been determined so far, and the structural biology of the majority of these RNA elements remains elusive. Over the past several years, various experimental techniques have been used to study the structures of IRESs, 3′ CITEs, ribosomal frame-shifting, and other translation-related RNAs and their protein complexes, which are summarized in Table 4 along with their advantages and limitations.

### 4.1. Biochemical Probing Methods

Historically, the characterization of RNA secondary structures has depended on enzymatic and chemical probing techniques, such as RNase digestion, DMS-probing, and SHAPE chemistry. Combined with covariation studies in natural isolates, these methods effectively identify base-pairing interactions and flexible regions within RNA molecules, identifying potential stems and loops in IRESs and CITEs and predicting long-range interactions [31,114]. An example of successful DMS-probing can be seen with earlier work on HCV IRES, where 18s rRNA was determined to bind in the unprotected, apical loop of HCV IRES IIId [114]. This was achieved by modifying the triple CCC region in 18s rRNA and using reverse transcription to show which positions are protected when bound to HCV IRES. DMS probing has also been used to predict secondary structure of the SARS-CoV-2 FSE [200], which is then further used for determining how antisense oligonucleotides change the conformation of FSE, and its effect on -1 frame shifts [200]. After the discovery of the SHAPE (selective 2′-hydroxyl acylation analyzed by primer extension) [220] method, secondary structures of many RNAs, including IRESs, 3′ CITEs, FSEs, and even whole genomes of select viruses, have been probed by this strategy [41,212,221,222,223]. For example, SHAPE reactivity has been used on various types of IRESs to determine flexible regions and their dynamic behavior [81,174,215,224,225]. For FMDV IRES, the changes in flexibility, measured by SHAPE reactivity, were combined with computer modeling software to show long-range interactions between domains IV and V of the 3′ UTR and the multiple stem-loop structures within the 5′ IRES [224]. However, probing data alone cannot reveal tertiary contacts or resolve complex folding patterns. Moreover, some RNA conformations are inherently dynamic or may only take certain structures in the presence of specific proteins; such transient or alternative conformations are challenging to characterize with static probing techniques that provide average signals of several conformations that can coexist in solution.

### 4.2. X-Ray Crystallography

While only a few studies report X-ray crystal structures of translation-related viral RNA domains, these high-resolution crystal structures of viral RNA domains have provided detailed insights into potential mechanisms of viral translation. Notably, the 2.84 Å resolution crystal structure of HAV IRES domain V, complexed with a synthetic antibody fragment, revealed a three-way junction scaffold held by an adenine-rich motif [195]. This structure uncovered a conserved architectural theme later identified in other picornavirus IRESs, such as EMCV J-K domain [65]. X-ray crystallography has also been used to solve structures of smaller motifs, such as the IIId domain of HCV IRES or junctions of dicistrovirus IRESs [174,175,204]. Other translation-related structures derived by X-ray crystallography were PTEs from SCV and PEMV2 RNAs, both in complex with a Fab chaperone [198,199]. These structures were topologically homologous, consistent with their similar binding to eIF4E. Subsequent electrophoretic mobility shift assays showed that a particular flipped-out guanine in both structures was critical for binding eIF4E and how these RNAs effectively mimic the mRNA 5′ cap to hijack eIF4E for viral translation [198,199]. Although a few are determined so far, the atomic models of translation-related RNAs show how particular loops and bulges interact with metal ions or fold into pseudoknots, pre-organizing the RNA for ribosome binding. An example of this is seen in the X-ray crystal structure of the frameshifting element found in SARS-CoV-2, where P1 and P2 Helices connect to loops 1–3 to form an H-type pseudoknot stabilized by cobalt(III)-hexamine, Mg^2+^, and K^+^ ions [164]. Nevertheless, studying full-length IRESs and 3′ CITEs with X-ray crystallography is difficult because they are often too large and flexible to form crystals. Even obtaining domain-level crystals requires extensive engineering, such as using antibody chaperones (as seen in the HAV IRES case) or removing flexible regions. Crystal structures show only one static conformation; many viral RNAs can adopt multiple structures depending on the context.

### 4.3. NMR Spectroscopy

NMR offers insights into the structure and dynamics of RNA elements, especially those under around 100 nucleotides. It has been used to detect local and global changes in IRES elements when they bind to ligands or proteins. For example, Davila-Calderón et al. [206] used NMR to determine the structure of the stem-loop II domain of the EV71 IRES both when free and when bound to a small-molecule ligand, DMA-135 [206]. They found that the binding of DMA-135 allosterically stabilized a conformation that recruits the host protein AUF1, thereby repressing viral translation. NMR has also helped model protein-RNA complexes. For instance, Dorn et al. [208] combined NMR, mass spectrometry, and small-angle X-ray scattering (SAXS) to generate an ensemble structure of the EMCV IRES domains D-F, which are located upstream of the core IRES bound to four RRMs of the ITAF PTBP1, and demonstrated that PTBP1 acts as an RNA chaperone, compacting and organizing the IRES. Structures of EMCV Domains J and K were also solved using NMR and shown to interact with the HEAT-1 domain of eIF4G through multiple interactions between the stem loop domains and bulges orchestrated by an A-rich pentaloop [65]. The secondary structure for the ribosome-binding structural element (RBSE) found in turnip crinkle virus (TCV) 3′ CITE was also determined by NMR; however, SAXS data were needed to generate its 3D molecular envelope and resolve the angles within the hairpin structures of RBSE [151]. The primary limitation of NMR is its size restriction: full IRES RNAs are often too large to analyze, so NMR typically focuses on individual domains or segments. It also requires isotope labeling and high concentrations, which can be challenging. The rapidly exchanging or flexible regions may not be well-resolved.

### 4.4. Cryo-Electron Microscopy (Cryo-EM)

Cryo-EM is an emerging technique for RNA structural determination, and it has been crucial for elucidating the structures of many proteins and protein-RNA complexes. It has significantly advanced the internal ribosome entry site (IRES) structural biology field by enabling detailed visualization of IRES-ribosome assemblies. For instance, Neupane et al. determined the structure of a CrPV IRES bound to the 40S ribosomal subunit at a 3.3 Å map resolution, revealing its intricate architecture that encircles the ribosomal head [211]. Recent cryo-EM investigations have captured interactions such as HCV IRES binding to eIF3-40S complexes and EMCV IRES engaging with initiation factors [101]. The 2023 study by Imai et al. employed cryo-EM to elucidate a dynamically regulated interaction between viral RNA and two distinct sites on eIF4G, providing insights into the mechanism by which EMCV IRESs recruit host factors [205]. The cryo-EM structure has also been solved for the complex between PCBP2 and domain IV of type I IRES found in poliovirus RNA [170]. Although determined at a resolution of 6.1 Å, the cryo-EM structure indicated that the GNRA tetraloop in domain IV does not directly interact with PCBP2, but the GNRA motif itself is essential for poliovirus translation, possibly through allosteric effects in modulating domain IV structure [170]. While cryo-EM excels in resolving large, asymmetric complexes, it necessitates homogeneous and stable samples. IRES-ribosome complexes often exhibit multiple conformational states, requiring sophisticated image classification techniques. Additionally, due to size limitations, the plant virus 3′ CITEs and smaller IRES elements have yet to be resolved using cryo-EM. One successful attempt at determining smaller structures is the cryo-EM structure of the SARS-CoV-2 FSE [41]. Although this cryo-EM structure is only at 6.9 Å resolution, it reveals the detailed ring formed by the three-stem pseudoknot of the FSE. Notably, cryoEM provides information from reconstituted complexes in vitro, which could differ from the RNA or RNA-protein complexes in infected cells. However, ongoing improvements in resolution and technology, such as cryo-tomography, are expected to enable atomic-level modeling of viral translation initiation complexes on the ribosome inside the cells.

## 5. Regulation of the Replication-Translation Switching

Because (+) sense RNA genomes serve as templates for two mutually exclusive processes, translation and replication, these viruses must regulate when and how their genomes are used for each process. Early in infection, translation dominates the production of viral proteins. Later, once sufficient viral polymerases build up, the viral RNA is redirected to replication [8,226]. Several mechanisms control this switch. For example, viral proteases cleave host initiation factors such as eIF4G during picornavirus infection. This cleavage suppresses host cap-dependent translation and enhances the relative efficiency of viral IRES-mediated initiation, reprogramming the host translation machinery for viral protein synthesis [227,228]. In poliovirus and enterovirus, the RNA elements play critical roles in regulating the translation-replication switching. For instance, although both domain I, which contributes to structural stability and replication initiation, and domain IV, which is crucial for translation initiation, can bind the same host factor PCBP2, the viral protease 3C or 3CD cleaves PCBP2 bound to domain IV, decreasing the translation and promoting replication [59,170,229,230]. Notably, domain I serve as a binding site for both viral proteins, like 3CD, and host proteins like PCBP2, facilitating the switch to replication by stabilizing RNA structures needed for VPg uridylation [231,232]. Conversely, viral replication complexes can physically sequester viral RNAs at membranes, blocking ribosome access. Some *cis*-acting elements have dual roles: a stem-loop may initially recruit the ribosome, but it may also help VPg uridylation and replication priming after polyprotein synthesis [13,190,233].

Differential expression of viral proteins can also regulate the balance between replication and translation processes [226]. Internal transcription of sub-genomic RNAs (in coronaviruses, alphaviruses) or frameshifting (in HIV, coronaviruses) modulates the ratio of structural to nonstructural proteins [234,235]. In flaviviruses, including HCV and related genera, translation and replication are tightly interconnected through long-range RNA rearrangements involving structured elements in the 5′ and 3′ UTRs. These rearrangements facilitate genome cyclization via complementary interactions between the 5′ and 3′ ends, supporting the assembly of the replication complex while also regulating translation by influencing ribosome access or initiation factor binding. Disruption of these UTR interactions can shift the balance between translation and replication, emphasizing their dual regulatory roles [175,236]. Additionally, the binding of viral or host proteins to IRES or CITE elements can be regulated by concentration or post-translational modifications [29,72,237]. Some viral ITAFs are produced only after infection, enhancing IRES activity [48]. For example, the stress protein DDX3 binds certain IRESs only under stress conditions, linking environmental cues to viral translation [238,239,240]. While this review focused on RNA components of the genome translation, viruses use a variety of structural RNA switches and factor engagements to coordinate the transition from translation to genome replication. A better understanding of the underpinnings of structural and mechanistic aspects of this crucial topic requires further investigation.

## 6. Perspectives and Future Directions

Despite significant progress in understanding the roles of RNA structures in translating (+) sense RNA viruses, several unanswered questions and ongoing challenges persist. Obtaining high-resolution structural information for large, dynamic viral RNA-protein complexes remains a significant challenge. While advances in cryo-EM offer new opportunities for such studies, further methodological improvements are necessary to fully clarify the intricate architectures of these assemblies. Regarding IRES-mediated translation, the specific roles of all the various ITAFs identified for different IRES types are not yet fully understood and need further research. In plant virology, dissecting the complex infection cycle within plant hosts and developing more robust experimental systems for biochemistry, genetics, and cellular biology are essential for a more comprehensive understanding of plant virus translation mechanisms. Moreover, many (+) sense RNA viruses contain multiple *cis*-acting elements within a single RNA molecule, and the functional interactions among these elements and the reasons for their multiplicity warrant further investigation.

The unique RNA structures and their interactions within positive-sense RNA viruses offer promising targets for developing new antiviral treatments. For example, the FSE in coronaviruses, vital for producing the viral RdRp, has attracted interest as a potential drug target. Blocking this frameshifting process has been shown to hinder viral replication, as seen in studies with SARS-CoV-2. This can be seen in previously mentioned cryo-EM structures, which have been applied in developing antisense oligonucleotides made of locked nucleic acids (LNAs) [41]. Likewise, targeting interactions between viral RNA structures, such as IRESs, and key host or viral proteins presents another effective strategy for stopping viral translation without significantly disrupting the host cell’s normal functions. An example of this can be seen in the previously mentioned NMR structures demonstrating conformational changes by DMA-135 that stabilize the AUF1-RNA complex and inhibit translation [206].

Future research in this field will likely focus on several key areas. Continued advancements in structural biology techniques, especially cryo-EM, will be vital for gaining more detailed structural insights into large and dynamic viral RNA-protein complexes. A deeper understanding of the complex host-virus interactions, including the roles of various host RNA-binding proteins (RBPs) and translation factors in modulating viral translation, will be crucial for developing targeted antiviral strategies. Exploring the similarities between viral and host mRNA translation mechanisms, particularly under conditions of cellular stress, could provide new insights into fundamental aspects of translation regulation and uncover novel antiviral targets. RNA-based therapeutics, which include strategies like antisense oligonucleotides and siRNAs targeting viral RNA structures, are also rapidly growing and have significant potential for fighting (+) sense RNA virus infections. Understanding the various RNA processing events in viruses and how they influence translation could also guide future therapeutic approaches. The idea of personalized medicine, where treatment is tailored to an individual’s genetic profile, might become increasingly relevant in RNA virus infections, leading to more effective and targeted therapies. Finally, the remarkable success of mRNA vaccine technology, especially in response to recent viral outbreaks, highlights the critical importance of a solid understanding of RNA virus translation and regulation for developing effective preventive measures.

## Figures and Tables

**Table 3:** Some crucial ITAFs and their regulatory roles in genome translation of (+) sense RNA viruses.

ITAF	IRESs	ITAF’s Roles	IRES Activities	References
PTB (hnRNP I)	PV, HRV	RNA chaperone and stabilizer; alters IRES secondary structure	Activation, inhibition (context-dependent)	References [56,70,172]
PCBP2 (hnRNP E)	PV, HAV	Binds IRES sites; restricts conformational flexibility; assists eIFs	Activation	References [168,169]
hnRNP A1	HRV-2	RNA chaperone; nucleocytoplasmic shuttling; affects structure	Activation, inhibition (context-dependent)	Reference [177]
La autoantigen	PV, HCV	Assists translation initiation; stabilizes IRES conformation	Activation, inhibition (HAV IRES)	References [57,178,179]
Unr	HRV	RNA chaperone; alters IRES secondary structure	Activation	Reference [58]
RACK1	HCV, DCV, CrPV	40S ribosomal subunit-associated protein, mediates IRES activity	Activation	References [180,181]
Gemin5	FMDV, HCV	Ribosome-associated protein, competes for ITAF binding	Inhibition	Reference [182]
Sam68	FMDV, EV71	Nucleocytoplasmic shuttling, stimulates IRES activity	Activation	References [82,183]

**Table 4:** Structures of translation-related RNAs (IRESs, 3′ CITEs and frameshifting elements) determined by various biochemical and biophysical methods.

Methods	Strengths	Limitations	Structures
X-ray Crystallography	Atomic-resolution 3D structures; precise protein-RNA contact sites	Requires crystal formation (difficult for large, flexible RNAs); static nature	PEMV2 PTE [199], SCV PTE [198], Donggang virus dumbbells [201] HAV IRES domain V [195], HCV IRES subdomains [174,202,203], IGR IRES [204]
Nuclear Magnetic Resonance (NMR)	Probes dynamic conformational changes; solution-state interactions; binding affinities	Size limitations for large complexes; spectral complexity	EMCV IRES + eIF4G/eIF4A complex [205], EV71 IRES SLII + DMA-135 [206], EMCV IRES J-K domain [65,207], PTBP1-EMCV IRES fragment [208], HCV IRES [209,210]
Cryo-Electron Microscopy (Cryo-EM)	High-resolution structures of large complexes; captures multiple conformational states	Requires large complexes; data processing complexity	HCV IRES-40S [101], CrPV IRES-40S [211], EMCV IRES-eIF4G/eIF4A [205]
Biochemical Methods	Maps RNA secondary structures in solution; identifies flexible regions; guides 3D modeling	Does not provide atomic resolution; mostly limited to secondary structure information	PTE structures [199,212], BTE 3′ CITE [213], Poliovirus IRES [214,215], HCV IRES [216], FMDV IRES [217], CrPV IRES [218], Coxsackievirus B3 IRES [219]

## Data Availability

No new data were created in this study. Data sharing is not applicable to this article.
